# Identifying Innate Resistance Hotspots for SARS-CoV-2 Antivirals Using In Silico Protein Techniques

**DOI:** 10.3390/genes14091699

**Published:** 2023-08-26

**Authors:** Stephanie Portelli, Ruby Heaton, David B. Ascher

**Affiliations:** 1 School of Chemistry and Molecular Biosciences, The University of Queensland, St Lucia, QLD 4072, Australia; 2 Baker Heart and Diabetes Institute, 75 Commercial Road, Melbourne, VIC 3004, Australia

**Keywords:** molnupiravir, ritonavir, nirmatrelvir, SARS-CoV-2, COVID-19, antiviral resistance

## Abstract

The development and approval of antivirals against SARS-CoV-2 has further equipped clinicians with treatment strategies against the COVID-19 pandemic, reducing deaths post-infection. Extensive clinical use of antivirals, however, can impart additional selective pressure, leading to the emergence of antiviral resistance. While we have previously characterized possible effects of circulating SARS-CoV-2 missense mutations on proteome function and stability, their direct effects on the novel antivirals remains unexplored. To address this, we have computationally calculated the consequences of mutations in the antiviral targets: RNA-dependent RNA polymerase and main protease, on target stability and interactions with their antiviral, nucleic acids, and other proteins. By analyzing circulating variants prior to antiviral approval, this work highlighted the inherent resistance potential of different genome regions. Namely, within the main protease binding site, missense mutations imparted a lower fitness cost, while the opposite was noted for the RNA-dependent RNA polymerase binding site. This suggests that resistance to nirmatrelvir/ritonavir combination treatment is more likely to occur and proliferate than that to molnupiravir. These insights are crucial both clinically in drug stewardship, and preclinically in the identification of less mutable targets for novel therapeutic design.

## 1. Introduction

Severe acute respiratory syndrome coronavirus 2 (SARS-CoV-2) is the virus responsible for coronavirus disease 2019 (COVID-19) [1]. COVID-19 has spread globally through respiratory transmission and was declared a pandemic by the World Health Organisation in March 2020 [2]. Since then, there have been over 670 million COVID-19 infections, resulting in almost 7 million deaths worldwide [3]. To mitigate the spread of this virus, vaccines [4], monoclonal antibodies [5], and antivirals [6] have been developed. 

The introduction of mutations within the SARS-CoV-2 genome has given rise to several lineages, of which the Omicron variant is currently prevalent [7]. Lineage emergence is typically mediated via the accumulation of mutations conferring a beneficial effect, such as the N501Y spike protein mutation, which increases viral transmissibility [8]. The consideration of such variation is crucial during novel therapeutic development, as it determines treatment longevity and efficacy [9]. 

Two antivirals have been approved for treatment of SARS-CoV-2: molnupiravir and nirmatrelvir/ritonavir (NMV/r). Molnupiravir is a ribonucleoside prodrug of N4-hydroxycytidine (NHC) that targets the SARS-CoV-2 RNA-dependent RNA polymerase complex (RdRp) [10]. The RdRp is responsible for viral genome transcription and is composed of non-structural proteins (NSP) 7, NSP8, and NSP12 [11], which binds molnupiravir. During viral replication, NHC, the active form of molnupiravir, is further phosphorylated intracellularly to produce the nucleotide analogue NHC triphosphate [12,13]. This is incorporated by RdRp as a substrate which results in the erroneous incorporation of guanosine or adenosine into the growing RNA strand, inducing an error catastrophe and, ultimately, viral death [12,13]. The NMV/r components nirmatrelvir and ritonavir, on the other hand, both target the NSP5 gene encoding the main protease (MPro) [14]. MPro is responsible for polyprotein cleavage, which mediates the assembly of viral replication machinery [15]. Both nirmatrelvir and ritonavir act as competitive inhibitors to polyproteins pp1a and pp1ab, thereby disrupting their cleavage and preventing further viral replication [14,15].

Despite promising results in clinical trials [12,14], a major concern with antiviral treatment is the development of resistance following prolonged selective pressure on the viral genome. Recent work has shed light on MPro mutations [16] and pathways [17] leading to resistance to nirmatrelvir, the newer component of the NMV/r drug combination. Towards the understanding of variation, we have previously characterized the effect of all possible missense mutations within the SARS-CoV-2 genome [18,19]. In doing so, we observed varying levels of purifying selection across the SARS-CoV-2 proteome, which helped identify genes encoding for helicase, NSP4, NSP9, ExoN, and, more interestingly, the RdRp as promising targets for antiviral development [18]. Furthermore, our previous work on drug resistance in *Mycobacterium* [20,21,22,23,24,25,26], *Acinetobacter baumannii* [27], and hepatitis C [28] utilized similarly characterized variant effects to identify resistance mechanisms. In this work, we apply a similar *ethos* on SARS-CoV-2 circulating variation prior to antiviral selective pressure to assess the potential of resistance development upon widespread clinical use. 

**Figure 1:**
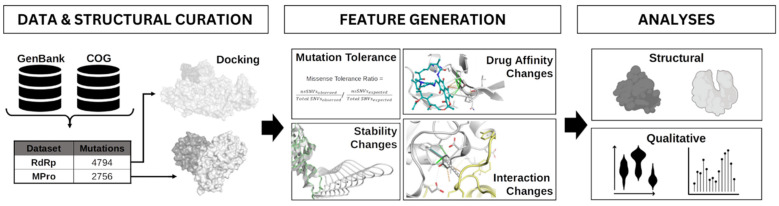
Workflow overview. Mutations were obtained from Genbank, COG, and the literature and were mapped to antiviral targets MPro and RdRp. Ligands were docked to their respective protein structures. Protein properties such as mutation tolerance, stability and binding partner interactions were generated. The effect of these mutations on these protein properties was investigated for putative resistance hotspots.

Specifically, we characterized the structural consequences of missense mutations on different protein properties, including stability and interactions with binding partners, and analyzed them in a gene-level, statistical, and structural context (Figure 1). Combining these aspects enabled us to identify variants having inherent resistance to either antiviral, which was more prominent for NMV/r than for molnupiravir. This work offers important clinical implications towards drug stewardship and resistance prevention, while also informing SARS-CoV-2 genomic surveillance. 

## 2. Materials and Methods

### 2.1. Data Curation

Initial curation of 179,931 genome sequences from GenBank [29] and the COVID-19 Genomics UK Consortium [30] permitted the alignment to the SARS-CoV-2 reference genome (NC_045512.2) to identify unique circulating missense mutations for all mature proteins including the antiviral targets, the RdRp complex (*n* = 4794), and MPro dimer (*n* = 2756; Table 1). For our analyses, we split the mutation data into high- and low-frequency mutations based on frequency distribution, where loci having a frequency under the 1st quartile (Q1) were considered as low frequency, while mutations with frequencies in the 4th quartile (Q4) we considered high frequency. 

### 2.2. Protein Curation

Experimental crystal structures of MPro (7SI9 [31]) and RdRp (7BV2 [32]) as biological assemblies were obtained from the RCSB Protein Data Bank [33]. These structures were then subjected to pre-processing in Maestro (Schrodinger suite, v. 2017-4) and Modeller [34] to remove water molecules beyond 5 Å of ligand binding and fill the missing atoms in these assemblies. The experimental structures were bound to the drugs nirmatrelvir (MPro) and remdesivir (RdRp), which were used to guide the docking of ritonavir and molnupiravir, respectively. In both cases, docking was carried out under standard parameters (Supplementary Table S1) in Glide (Schrodinger suite, v. 2017-4), using chemical structures obtained from PubChem and prepared in LigPrep (Schrodinger suite, v. 2017-4). Docking of missing ligands was carried out to obtain separate protein structures bound to the antivirals in this study, which were required inputs for mmCSM-lig [35] to calculate the effects of circulating variants on ligand affinity. The resultant docked ligands occupied similar orientations to their reference ligands, at binding free energies ranging from –6.17 kcal/mol to –7.68 kcal/mol (Supplementary Figure S1). Despite the often limiting assumptions introduced by docked poses compared to experimental co-crystallized ligands, these resultant pose properties were consistent with prior work and appropriate for our subsequent calculations. 

### 2.3. Mutational Tolerance

The mutational tolerance of MPro and RdRp was calculated for the NSP5, NSP7, NSP8, and NSP12 sequences on a per-gene and per-residue basis using the missense tolerance ratio (MTR) [18,36,37] in R. The MTR score represents the ratio between the observed proportion of missense mutations compared to synonymous mutations against the same proportion of expected mutations within a residue window surrounding a residue or considering the whole gene (Equation (1)). Negative MTR values represent residues under negative selection, whereas a score of 1 represents residues in neutral selection and positive values represent residues under positive selection. Whilst calculating the residue level MTR, a sliding window of 21 residues centred around the residue of interest accounted for mutational tolerance counts. The score is calculated based on mutation information from a population and can, therefore, act as a measure of deleteriousness across a specific gene or gene region.

(1) $$MTR\;=\;\frac{\frac{nonsynonymous\;SNPs_{observed}}{Total\;SNPs_{observed}}}{\frac{nonsynonymous\;SNPs_{\exp ected}}{Total\;SNPs_{\exp ected}}}$$

### 2.4. In Silico Mutation Characterization 

The effects of all mutations on protein stability (DynaMut2) [38] and interaction affinity with other proteins (mmCSM-PPI) [39], ligands (mmCSM-lig) [35], and nucleic acids (mCSM-NA) [40] were calculated using *in silico* biophysical tools that rely on graph-based signatures accounting for mutant environment. Mutation distances to different interaction sites were also generated to filter ligand, nucleic acid, and protein–protein affinity values to those mutations which occurred within 10 Å. These filtered values were used to assess the overall effects of high-frequency mutations on the protein, as a proxy for fitness. As MPro is a homodimer, with duplicate mutations across both chains, values for effects of mutations were calculated solely on chain A. However, as NMV/r binding occurs at the interface, the changes in ligand affinity to either nirmatrelvir or ritonavir analyzed were those closest to ligand binding, which, at times, occurred on chain B.

### 2.5. Qualitative Analyses

High-frequency mutations were subjected to a qualitative analysis, as previously described in tuberculosis [20]. Briefly, the data were categorized based on mutational effect on the ΔΔG threshold: those which imparted a negligible effect (+/−0.05 kcal/mol), a mild effect (+/−0.05–+/−0.5 kcal/mol), a moderate effect (+/−0.5–+/−1kcal/mol), or a large effect (>+/−1 kcal/mol). Negligible effects were considered neutral, while other effects were prioritized according to interacting molecule size, as follows: ligand affinity, nucleic acid affinity, protein–protein interaction affinity, and overall protein stability. 

Finally, these effects, along with auxiliary features describing mutation environment were also statistically compared between high-frequency and low-frequency mutations using an unpaired two-sided *t*-test (R Studio, v. 1.4.1717). Auxiliary features included residue depth and relative solvent accessibility (RSA) generated using Biopython [41], along with residue-level interaction counts forged by wildtype residues at mutation sites, and their changes upon mutation, calculated using Arpeggio [42]. 

## 3. Results 

### 3.1. Mutations Distributed across the Full Gene Targets

**Figure 2:**
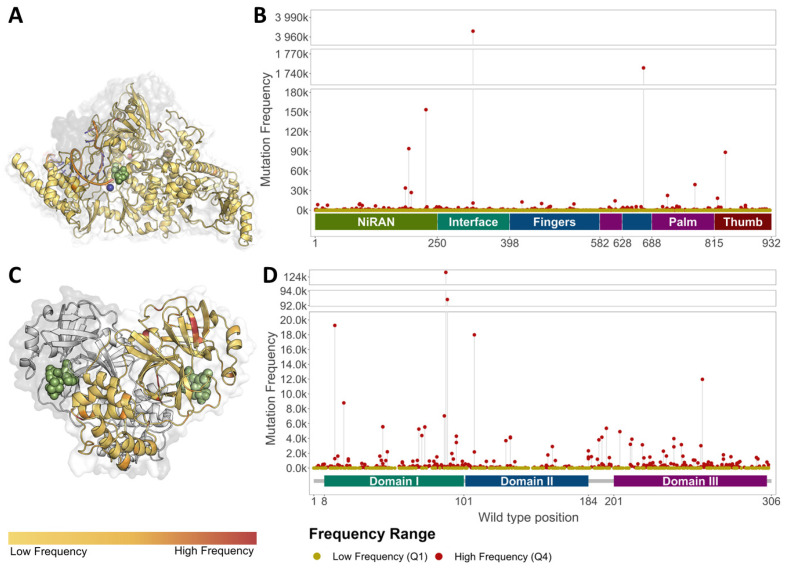
Distribution of Missense mutations across antiviral targets. Low-(yellow) and high-(red) frequency mutations across NSP12 within the RdRp (**A**,**B**), and MPro (**C**,**D**). Extremely-high-frequency mutations were observed close to NMV/r binding within MPro ((**C**); Supplementary Figure S2A), as compared to molnupiravir binding in the RdRp ((**A**); Supplementary Figure S2B–D). Ligands rendered as green spheres, while Mg^2+^ and Zn^2+^ ions in the RdRp are rendered as blue and red spheres, respectively.

Circulating genetic variation compiled prior to antiviral approval highlighted that, over the course of 18 months, every residue across targets MPro (NSP5) and RdRp (NSP 7, 8, 12) was subject to variation. A total of *n =* 2756 missense mutations were observed for the MPro dimer, while *n =* 4794 were observed for the RdRp complex (Table 1; Figure 2, Supplementary Figure S2). Considering absolute frequencies, our MPro low-frequency mutations all had a density of 1, while high-frequency mutations had a count ranging from 45 to 124,622. Almost a third (27.2%) of the high-frequency mutations had a prevalence below 100, while 17.4% were above 1000, with a median lying at 213 (Supplementary Table S2). When considering data available for the RdRp complex, mutation densities across genes NSP7, NSP8, and NSP12 within the low-frequency group were comparable to those in MPro and ranged from 1 to 2 (mean: 1.12). Densities within the high-frequency group ranged from 50 to 3,968,858, which was broader than that observed in MPro. However, the median observed for RdRp was lower, sitting at 175. This suggests that although certain unique residues had a higher frequency in RdRp, overall, MPro had a higher proportion of high-frequency mutations (Supplementary Table S2). Notably, across the targets, the distributions of the medium-frequency mutations (Q2–Q3) had similar ranges, between 2 and 50 (Supplementary Figure S3).

### 3.2. MPro and the RdRp Had Different Levels of Gene Mutational Tolerance

To further assess the likelihood of target gene innate resistance to the approved antivirals, we generated the likelihood of genes NSP5, NSP7, NSP8, and NSP12 to become enriched in missense mutations compared to their synonymous counterparts, through the missense tolerance ratio [18,36,37]. 

An initial comparison of the target gene-level mutation tolerances with non-target SARS-CoV-2 genes revealed that genes NSP5 and NSP12 have a lower tolerance to accumulation of missense mutation, indicating that they are under strong purifying selection [18]. As missense mutations have been observed to cause drug resistance in other infectious diseases like tuberculosis [43], particularly in the absence of horizontal gene transfer, this purifying selection suggests that the target genes present an inherently lower opportunity for antiviral resistance to eventually emerge. 

**Figure 3:**
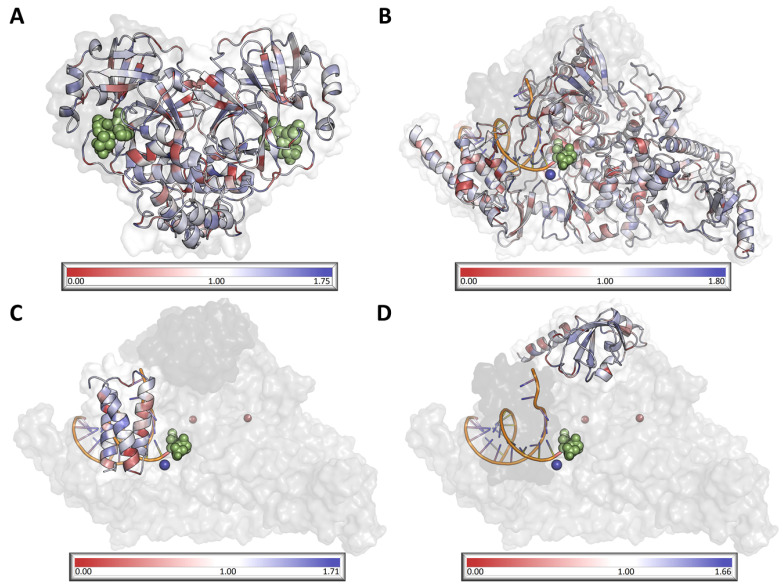
Visualisation of mutation tolerance in SARS-CoV-2 MPro dimer (**A**) and RdRp NSP12 (**B**), NSP7 (**C**), and NSP8 (**D**). All protein chains are colored according to their respective MTR score, where red indicates residues under negative selection, white indicates residues under neutral selection, and blue indicates that the residue is under positive selection. Ligands are represented as green spheres, while magnesium and zinc ions within the RdRp are represented in dark blue and dark red, respectively.

When considering both targets, NSP12 was observed to be less tolerant than NSP5, suggesting that eventual resistance to molnupiravir is less likely to develop than to NMV/r (Figure 3, Supplementary Table S3). In observing residue-level scores for both targets, general statistics (Supplementary Table S3) indicate a large range in values across each protein, where regions were observed under positive selection, suggesting allowance of mutation accumulation, while most residues across both proteins were observed to be under neutral selection (MTR = 1; Supplementary Table S3).

### 3.3. Molecular Drivers of Mutation Retention

Given the extent to which the gene targets were mutated, and their inherent tolerance to this mutation, we next determined what factors lead to different mutation frequencies, and the subsequent establishment of mutations within a population. High-frequency (4th quartile) and low-frequency (1st quartile) mutations were extracted from our dataset, and their effects on protein stability, dynamics, and affinity to other proteins, nucleic acids, and antivirals were generated. Our previous work on tuberculosis [20] mutations identified a link between resistance mutation frequency and the extent of effects imparted. Specifically, in tuberculosis drug targets, resistance mutations with lower molecular impact were observed to occur at higher frequencies, suggesting that fitness can be estimated based on in silico measures [20]. This finding served as a basis for a statistical comparison between the effects imparted by SARS-CoV-2 high- and low-frequency mutations, as a measure of possible resistance development. 

MPro low-frequency mutations localized at more buried residues (RSA *p*-value: <0.0001; residue depth *p*-value: <0.0001) and were observed to cause larger destabilizing effects (DynaMut2 *p*-value: <0.0001) than high-frequency mutations. When considering the effects on protein interactions, low-frequency mutations resulted in larger decreases in homodimer (mmCSM-PPI *p*-value: <0.0001) and ligand affinities (mmCSM-lig *p*-values: <0.01), while also localizing closer to ligand binding (*p*-values < 0.0001), and to the protein–protein interface (*p*-value < 0.001; Supplementary Table S4). These effect profiles suggest that lower-frequency mutations tend to impart a higher fitness cost to the target homodimer, MPro, a pattern also observed in tuberculosis resistance mutations [20].

Mutations in NSP12, which binds molnupiravir within the RdRp, followed similar patterns. Specifically, the low-frequency group localized closer to the protein core (RSA *p*-value < 0.0001, residue depth *p*-value < 0.0001), molnupiravir binding (*p*-value < 0.0001), and the nucleic acid (*p*-value < 0.001), and protein–protein interfaces (*p*-value < 0.05) than higher-frequency ones. Within these loci, lower-frequency mutations also lead to more considerable reductions in protein stability (Dynamut2 *p*-value < 0.0001) and protein–protein affinity (*p*-value < 0.0001), and increases in nucleic acid affinity (*p*-value < 0.0001; Supplementary Table S5). Similar effects on protein–protein interactions have been observed as a result of *M. tuberculosis* rifampicin-resistant mutations, however, opposite effects on nucleic acid affinity were observed [20]. While this discrepancy can be explained through the contrasting molnupiravir and rifampicin modes of action, the resistance potential of low-frequency mutations in the RdRp appears to be lower than that within MPro, especially when considering that lower-frequency mutations, in spite of their lower observed frequency, offer less drastic protein fitness effects. 

### 3.4. Effects of High-Frequency Mutations across the Functional Protein Complex

While lower-frequency mutations were observed to be more detrimental, we assessed the effects of high-frequency mutations, due to their higher likelihood of innate resistance development. When considering the combined effects of all high-frequency mutations observed in MPro (*n =* 345), 49.3% of mutations primarily lead to protein destabilization, while 19.4% directly reduced ligand affinity (mmCSM-lig; 17.4% for nirmatrelvir, 2.0% for ritonavir) and 14.8% affected dimer stability (mCSM-PPI2). No high-frequency mutations were observed to increase affinity to either ligand, while some were observed to increase protein (10.4%) and complex (4.1%) stabilities. Notably, a further 2.0% (*n* = 7) was observed to confer overall mild effects, which, in our previous work on tuberculosis drug-resistance mutations, had higher frequencies [20]. The frequencies of this mild cohort ranged from 55 to 5376, meaning that just under half of these mutations (42.9%): Q69R, D248E, and T196M, had higher frequencies than the median (213; Supplementary Table S2). This suggests that these mutations may facilitate resistance development through compensatory effects, similar to what was observed in tuberculosis resistance [20]. 

On the other hand, when analyzing the overall effect of high-frequency mutations in the RdRp *(n =* 1119)*,* we noticed that, while most (47.9%) of mutations caused protein destabilization, direct disruptions in affinity to molnupiravir only accounted for 2.8% of mutations. Instead, similar to what has been observed when analyzing tuberculosis DNA-dependent RNA polymerase resistance mutations, some mutations reduced overall complex stability (20.9%), but, unlike what was observed in the same study, decreases in nucleic acid affinity, which would directly interfere with the molnupiravir mode of action, were limited to 1.2% of mutations. In contrast, 6.0% of mutations lead to an increase in nucleic acid affinity, while 0.08% (*n* = 1) lead to an increase in molnupiravir affinity—both of which can be beneficial upon widespread molnupiravir use. Finally, 1.5% of mutations (*n* = 18) were observed to confer mild effects, whose frequencies ranged from 61 to 1736. Of these, 44.4% had higher frequencies than the median (175; Supplementary Table S2): D258Y, M174I, N743S, I223M, M615I, P178H, N177S, and T226M. While this still implies that such mutations can have compensatory effects enabling resistance, the drug-naïve frequencies observed suggest that this risk is lower than that for MPro mild mutations.

### 3.5. Effects of High-Frequency Mutations across the Antiviral Binding Site

To further assess the mechanisms imparted by mutations with respect to innate resistance development, we carried out a similar analysis on high-frequency mutations localized at the drug-binding sites of our targets, which was considered to include residues within 10 Å of drug binding. Within MPro, 17.7% (*n =* 61 for either ligand) of high-frequency mutations occurred within 10 Å of nirmatrelvir or ritonavir binding. Of these, 61.8% of mutations decreased protein stability (DynaMut2; mildly: 14.7%; moderately: 30.9%; highly: 16.2%) while 29.4% increased protein stability (mildly: 25.0%; moderately: 4.4%). Some mutations at the binding site also occurred at the protein–protein interaction surface (32.4%), and 54.5% of which (17.6% of all binding site mutations) caused mild reductions in dimer stability. Due to nirmatrelvir and ritonavir localized binding, mutation subsets within 10 Å of either ligand differed minimally (9.8%) amongst themselves. All mutations within interaction distance of either ligand greatly reduced the affinity to the antiviral. When comparing the effects of overlapping mutations (*n =* 55) on affinity to either ligand, we observed that 98.2% (*n =* 54) of mutations preferentially reduced nirmatrelvir binding affinity to a greater extent than that to ritonavir. This is similar to other smaller-scale studies [16] and corroborates the notion that, even though nirmatrelvir is the newer drug within this SARS-CoV-2-specific combination, it does not offer any advantage over ritonavir in terms of innate resistance protection from MPro drug-naïve circulating mutations. 

On the other hand, within the RdRp, only 2.9% (*n =* 35) of high-frequency mutations localized within 10 Å of molnupiravir binding. These mutations were only present in NSP12 and amounted to 3.6% of high-frequency mutations within this subunit. Of these binding site mutations, 91.4% decreased protein stability (DynaMut2; mildly: 5.7%; moderately: 42.9%; highly: 42.9%) while 5.7% mildly increased protein stability. As the RdRp consisted of three subunits, some binding site mutations were also present within 10 Å of other proteins (57.1%) or the nucleic acid transcript (22.9%). Interestingly, while most effects on protein–protein affinity observed from this subset are mild (45.7%), most binding site mutations (14.3%) within interaction distance of nucleic acids drastically increased affinity. Considering that molnupiravir requires affinity to nucleic acids to act as a nucleic acid analogue, molnupiravir-naïve circulating mutations at the active site likely enhance the mode of action of the antiviral. When noting the direct effect of binding site mutations on molnupiravir affinity, 77.1% of mutations cause mild reductions in affinity, while 17.1% reduce affinity moderately. This suggests that, even though circulating mutations already affect affinity, the risk of resistance development remains lower than that observed for NMV/r.

### 3.6. Antiviral Binding Sites Were More Enriched in Low-Frequency Mutations

**Figure 4:**
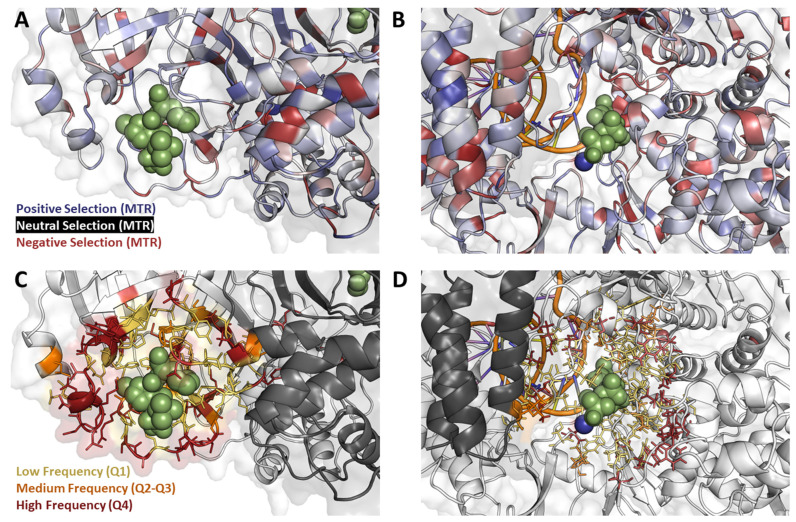
Mutation Properties at the binding site. Mutations located within 10 Å of ligand binding mapped onto MPro (**A**,**C**) and RdRp (**B**,**D**) show different levels of mutation tolerance across targets (**A**,**B**), as well as different frequencies (**C**,**D**). The residues surrounding ritonavir and nirmatrelvir binding site are under positive selection (**A**), meaning they are more tolerant to mutation accumulation when compared to the greater number of residues under negative selection in the molnupiravir binding site (**B**). Meanwhile, the RdRp binding site has a greater number of high-frequency mutations (**D**) when compared to the binding site of MPro (**C**).

Examining the antiviral binding sites more closely also revealed that 25.9% (*n* = 357) of all the MPro mutations localized within the active site, most occurred in, but were not limited to, the chain bound to the drug (A: 93.0%; B: 7.0%). Across the RdRp, however, only 0.05% (*n* = 247) of the total mutations were localized within molnupiravir binding, and were mostly localized within NSP12 (98.4%; *n* = 243), with minimal occurrence within NSP7 (Table 1; Figure 4C,D).

Generally, it was observed that distribution of mutation frequencies was different to that expected by normal distribution. When considering the MPro dimer, while most binding site mutations 51.5% (*n* = 184) lay in the interquartile range, 29.6% (*n* = 106) were low-frequency and only 18.7% (*n* = 67) were high-frequency. Similar patterns were observed for the RdRp, where 48.2% (*n* = 119) lay within the interquartile range, 37.7% (*n* = 93) were low-frequency, and only 14.2% (*n* = 35) were high-frequency.

While the frequencies at the direct binding site suggest low risk of innate resistance, the presence of mutations, whether high- or low-frequency, at close proximity to antiviral binding could still pose a risk of resistance emergence when selective pressure is applied. Of note, the observed antiviral-naïve mutation frequency distribution, coupled with the mild effects observed when analyzing some high-frequency mutations, suggests possible compensatory epistasis development, similar to what has been observed in *M. tuberculosis* [20]. 

### 3.7. Mutational Tolerance Patterns at the Antiviral Binding Sites Highlight Different Inherent Resistance Propensities

Finally, to fully assess the variables attributing to potential innate resistance at the antiviral binding site, we analyzed the mutation tolerance patterns, using MTR [18,36,37], within this region. Specifically, we found that the NMV/r binding site at the MPro homodimer interface was relatively tolerant to mutation (Figure 4A; blue), having a mean MTR score of 1.08. In practice, this implies that any emerging mutations at this site would impart a minimal fitness cost to protein thermodynamics, suggesting a higher propensity for resistance development under widespread antiviral usage. At the molnupiravir binding site within NSP12, on the other hand, we observed a lower tolerance to missense mutation (Figure 4B; red), where binding site residues had a mean MTR score of 0.45.

Mutations that occurred at a high frequency close to the NMV/r binding site were associated with large decreases in drug-binding affinity (ΔΔG < −2.00 kcal/mol; Figure 4C). Considering the observed high mutational tolerance at this MPro region, NMV/r affinity-disrupting mutations do not seem to confer large fitness costs on the overall folding and function of the protein. This again suggests that, given the high-frequency circulating mutations, there is a high risk for NMV/r resistance to develop as the antiviral becomes more routinely used. 

In contrast, the high-frequency mutations located near the molnupiravir binding site in RdRp had milder effects on ligand binding affinity (ΔΔG < −1 kcal/mol; Figure 4D). Paired with the lower mutational tolerance, this suggests that the introduction of affinity-disrupting mutations to this binding site is associated with a fitness penalty and, hence, is less likely to occur. This, in turn, reduces the chances for resistance development upon repeated antiviral exposure. 

Compared to MPro, the RdRp has a more essential role in viral replication and survival. Consequently, any mutations which accumulate in NSP12 and, possibly, contributing to resistance are under greater purifying selection than observed in the NMV/r binding site, as there may confer larger fitness costs. These findings, congruent with our other findings, suggest that resistance upon widespread clinical use is less likely to develop against molnupiravir than it is for NMV/r. 

## 4. Discussion

The emergence of SARS-CoV-2 in 2020 led to the largest, modern-day health emergency faced by the global population. While the state of emergency for the pandemic has officially been revoked in May 2023, COVID-19 remains a highly infectious and potentially lethal pathogen. Because of this, appropriate stewardship of the novel antivirals is crucial to prevent further disease prevalence due to drug resistance emergence. 

Early in the pandemic [18], we had identified regions across the SARS-CoV-2 genome tolerant to missense mutation accumulation, in the absence of antiviral selective pressure. These regions can be considered as potential “resistance hotspots”, as they can accommodate the introduction of resistance-causing mutations. That work hinted at the inherent inadequacy of MPro, and the potential for NSP12 as robust drug targets, from a genetic variation tolerance standpoint [18]. This work builds upon that initial knowledge and investigates the effect of genetic variation on the now-approved antivirals. Using updated, but drug-naïve, genetic variation, this work established that the NMV/r binding site in MPro had a higher mutational tolerance and a greater proportion of low-frequency mutations when compared to the molnupiravir-binding site in RdRp. Notably, even at the whole-gene level, MPro exhibited a greater tolerance to missense mutation than RdRp, implying that mutations which lead to drug resistance are more likely to accumulate. Prior research on resistance in viruses found that increased mutation rates, as those implied for MPro through increased tolerance, lead to an increase in resistance development [44,45].

Next, we explored the molecular mechanisms underpinning the observed mutation frequencies, and found that, statistically, low-frequency mutations in RdRp and MPro had significantly greater effects on protein properties, suggesting detrimental effects on viral and protein fitness. Previous knowledge on other organisms indicates that mutations which lead to drug resistance typically exert a negative effect on the protein [20,43]. In *M. tuberculosis*, this was particularly the case for lower-frequency mutations [20], and similar patterns were observed for SARS-CoV-2 variants in this study. While milder effects on protein function have been observed for high-frequency mutations, these variants tend to be more transmissible [46], meaning that they are more likely to be exposed to antiviral selective pressure, and still lead to resistance.

To address this possibility, we also assessed the effects of high-frequency mutations in a combined manner and found that most mutations across either target primarily lead to protein destabilization. Notably, however, the proportion of mutations which directly reduced antiviral affinity was higher for MPro than for the RdRp, further suggesting that, even when considering ‘less detrimental’ high-frequency mutations, the risk of innate resistance is higher for MPro than for RdRp. 

Towards highlighting the risk of innate resistance across MPro and RdRp, this work identified the susceptibility to, and consequential effect of, missense mutations within these antiviral targets. By predicting the effects of circulating variation computationally, it was possible to identify general trends linked to resistance development. Collectively, our findings strongly indicate that NMV/r has a greater risk of encouraging potentially drug-resistant mutation development upon widespread use. Notably, our findings offer a robust theoretical foundation for assessing resistance development, which would greatly benefit from experimental validation prior to clinical application. Practically, this information would be especially useful to guide drug stewardship efforts, so that resistance development can be delayed as much as possible. Further to that, similar approaches can be applied to assess resistance to novel antivirals, while also helping guide resistance-resistant drug discovery. 

## Figures and Tables

**Table 1:** Mutation distribution densities across RdRp and MPro antiviral targets. Considering unique genes across the target complexes, NSP12 harboured the highest proportion of mutations, however, NSP5 monomers in MPro harboured a higher number of mutations within interaction distance of ligand binding.

Target	Gene	Mutations Per Gene	High- Frequency Mutations	Low- Frequency Mutations	Mutations within 10 Å of Ligand Binding
MPro	*NSP5*	1378	345	345	357
RdRp	*NSP7*	299	1199	1199	247
	*NSP8*	560			
	*NSP12*	3935			

## Data Availability

This work used publicly available amalgamated data, and no ethics approval was required.

## Supplementary Materials

The following supporting information can be downloaded at: https://www.mdpi.com/article/10.3390/genes14091699/s1, Table S1: Parameters used for ligand docking; Table S2: Mutation frequency statistics across antiviral targets; Table S3: Mutational Tolerance Statistics across antiviral targets; Table S4: Significant In silico features distinguishing MPro high and low frequency mutations; Table S5: Significant in silico features distinguishing NSP12 high and low frequency mutations; Figure S1: Antiviral docking modalities; Figure S2: Mutation frequency distribution across gene targets; Figure S3: Distribution of Medium Frequency mutations across genes.
