# Supplementary material for: Identifying Innate Resistance Hotspots for SARS-CoV-2 Antivirals Using In Silico Protein Techniques
Source: Genes (Basel). 2023 Aug 26;14(9):1699. doi: 10.3390/genes14091699 (PMC10531314; doi:10.3390/genes14091699)
Supplement: Supplementary file 1 [file genes-14-01699-s001.zip › genes-2523014-supplementary.pdf]

Supporting Information

Identifying innate resistance hotspots for SARS-CoV-2 antivirals using in silico protein techniques

Stephanie Portelli[1,2*#], Ruby Heaton[1*] and David B. Ascher[1,2#]

1 School of Chemistry and Molecular Biosciences, The University of Queensland, St Lucia, Queensland, Australia

2 Baker Heart and Diabetes Institute, 75 Commercial Road, Melbourne, Victoria, Australia

[*]These authors contributed equally to the manuscript

[#]Correspondence should be addressed to D.B.A. on Tel: +61 7 336 53991 and Email: d.ascher@uq.edu.au. Correspondence may also be addressed to S.P. s.portelli@uq.edu.au.

Supplementary Table S1. Parameters used for ligand docking. Final ligand docking parameters for ritonavir in MPro (PDB: 7SI9) chains A and B, and molnupiravir in the NSP12 subunit of the RdRp (PDB: 7BV2). Initial parameters tested a grid size of 20Å x 20Å x 20Å, which was increased to 25Å x 25Å x 25Å for docking ritonavir in chain B as initial size yielded no poses.

**Ligand**	**Ritonavir, chain A**	**Ritonavir, chain B**	**Molnupiravir**
**x-coordinate**	-7.88	-44.67	95.85
**y-coordinate**	40.30	40.30	92.86
**z-coordinate**	-19.31	19.31	103.09
**Grid size**	20Å x 20Å x 20Å	25Å x 25Å x 25Å	20Å x 20Å x 20Å
**Binding free energy (kcal/mol)**	-6.24	-7.68	-6.17

Supplementary Table S2. Mutation frequency statistics across antiviral targets. While the absolute mutation frequency observed was highest within the RdRp, higher densities were observed within MPro high frequency groups, as highlighted through the Median values, suggesting higher propensity of innate resistance.

**Mutation group**	**Minimum**	**Maximum**	**Median**	**Mean**
MPro Low	1	2	1	1.04
MPro High	45	124622	213	1401.02
RdRp Low	1	2	1	1.12
RdRp High	50	3968858	175	5809.30

Supplementary Table S3. Mutational Tolerance Statistics across antiviral targets. Mutational Tolerance of SARS-CoV-2 MPro (NSP5) and RdRp (NSP7, NSP7, NSP12) genes.

Gene	Gene-level MTR	Residue-level Mean	Residue-level Mode	Residue-level Minimum	Residue-level Maximum
**NSP5**	0.79	0.97	1	0	1.75
**NSP7**	0.76	0.91	1	0	1.71
**NSP8**	0.72	0.96	1	0	1.66
**NSP12**	0.74	0.94	1	0	1.80

Supplementary Table S4. Significant In silico features distinguishing MPro high and low frequency mutations. The Welch Two-Sample t-test identified that mutation retention within MPro was reliant on different features, including mutation localisation, effects on protein stability and affinity to interacting MPro monomer, hydrogen and polar bond networks, amongst others. These mutations were also observed to distribute significantly differently with respect to antiviral binding, affecting antiviral affinity to significantly different extents.

Feature	Mean High Frequency	Mean Low Frequency	*p*-value
**Proximal Bonds count (Wildtype)**	62.29	76.43	6.81E-09
**Distance to Nirmatrelvir (Å)**	18.26	15.08	3.36E-07
**Distance to Ritonavir (Å)**	17.60	14.67	7.34E-07
**Change in Protein-Protein Affinity**	-0.05	-0.17	1.69E-06
**Change in Hydrogen Bonds count (Mutant)**	0.16	0.54	2.17E-06
**Hydrogen Bonds count (Wildtype)**	1.75	2.23	9.51E-06
**Relative Solvent Accessibility**	41.59	28.63	1.18E-05
**Residue Depth**	2.40	2.89	1.26E-05
**Change in Protein Stability (DynaMut2)**	-0.48	-0.78	1.42E-05
**van der Waals Clashes (Wildtype)**	1.83	2.33	1.78E-05
**Polar Bonds count (Wildtype)**	2.50	3.10	5.18E-05
**Change in Polar Bonds counts (Mutant)**	0.26	0.63	2.24E-04
**Change in van der Waals Clashes (Mutant)**	-0.34	-1.58	2.65E-04

**PI-PI Interaction count (Wildtype)**	0.03	0.11	4.26E-04
**van der Waals Forces (Wildtype)**	0.86	1.13	6.66E-04
**Change in Nirmatrelvir affinity**	-0.46	-0.73	1.16E-03
**Distance to Protein-Protein Interface (Å)**	14.62	12.99	1.54E-03
**Carbon-PI Bond count (Wildtype)**	0.20	0.36	3.51E-03
**Change in Ritonavir Affinity**	-0.37	-0.53	8.16E-03
**Change in Ionic Bond count (Mutant)**	0.02	0.21	8.44E-03
**Change in Weak Polar Bond count (Mutant)**	-0.16	-0.54	8.85E-03
**Aromatic Bond count (Wildtype)**	0.07	0.35	1.22E-02
**Weak Polar Bond count (Wildtype)**	1.33	1.57	1.65E-02
**Ionic Bonds count (Wildtype)**	0.08	0.22	2.61E-02
**Change in Carbonyl Bond count (Mutant)**	0.05	0.00	3.09E-02
**Change in PI-PI Interactions count (Mutant)**	-0.02	0.04	3.66E-02

Supplementary Table S5. Significant in silico features distinguishing NSP12 high and low frequency mutations. The Welch Two-Sample t-test highlighted that within NSP12, mechanisms of retention included mutation localisation, as well as effects on protein stability and interaction affinities to other RdRp subunits (NSP7 and NSP8) and nucleic acids. Notably, non-polar interaction networks were more distinctly affected by the mutation subsets analysed, as opposed to polar ones observed in MPro. Considering molnupiravir binding, significantly distinct distributions were observed with respect to mutation localisation, as well as effects on ligand affinity between frequencies.

Feature	Mean High Frequency	Mean Low Frequency	*p*-value
**Relative Solvent Accessibility**	38.09	23.14	8.00E-26
**Protein-Protein Affinity**	-0.05	-0.16	1.09E-18
**Residue Depth**	2.43	2.92	9.05E-14
**Change in Protein Stability (DynaMut2)**	-0.85	-1.13	1.08E-12
**Distance to Molnupiravir (Å)**	21.46	19.29	6.63E-10
**Change in Nucleic Acid Affinity**	0.23	0.97	8.05E-09
**Change in Molnupiravir Affinity**	-0.02	-0.05	3.61E-05
**Distance to Nucleic Acid (Å)**	27.76	25.42	2.57E-04
**Proximal Bond count (Wildtype)**	71.09	74.99	2.88E-03
**Aromatic Bond count (Wildtype)**	0.38	0.57	1.63E-02
**Metal Bond count (Wildtype)**	0	0.01	3.20E-02
**Change in Amide-Amide Bond count (Mutant)**	0.01	-0.02	3.21E-02
**Change in Metal Bond count (Mutant)**	0	0.01	3.44E-02
**Change in Carbon-PI Bond count (Mutant)**	0.03	0.08	3.78E-02
**Distance to Protein-Protein Interface (Å)**	19.01	17.72	4.09E-02

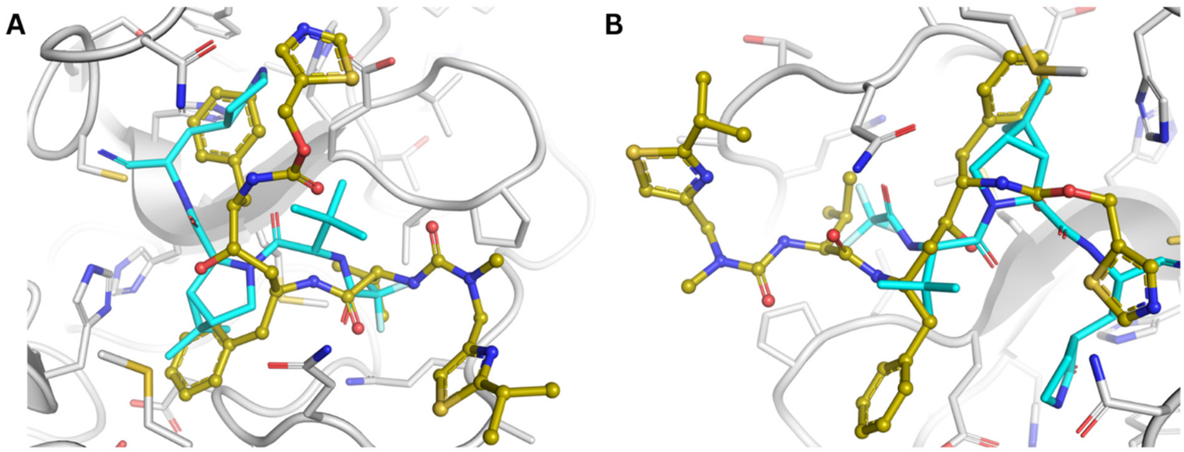

Supplementary Figure S1. Antiviral docking modalities. Antivirals molnupiravir (A) and ritonavir (B) docked to the NSP12 component of the RdRp and MPro respectively. The docked pose of molnupiravir (olive; A) is comparable with remdesivir (cyan). The pose of docked ritonavir (olive; B) is comparable to the co-crystallized nirmatrelvir (cyan).

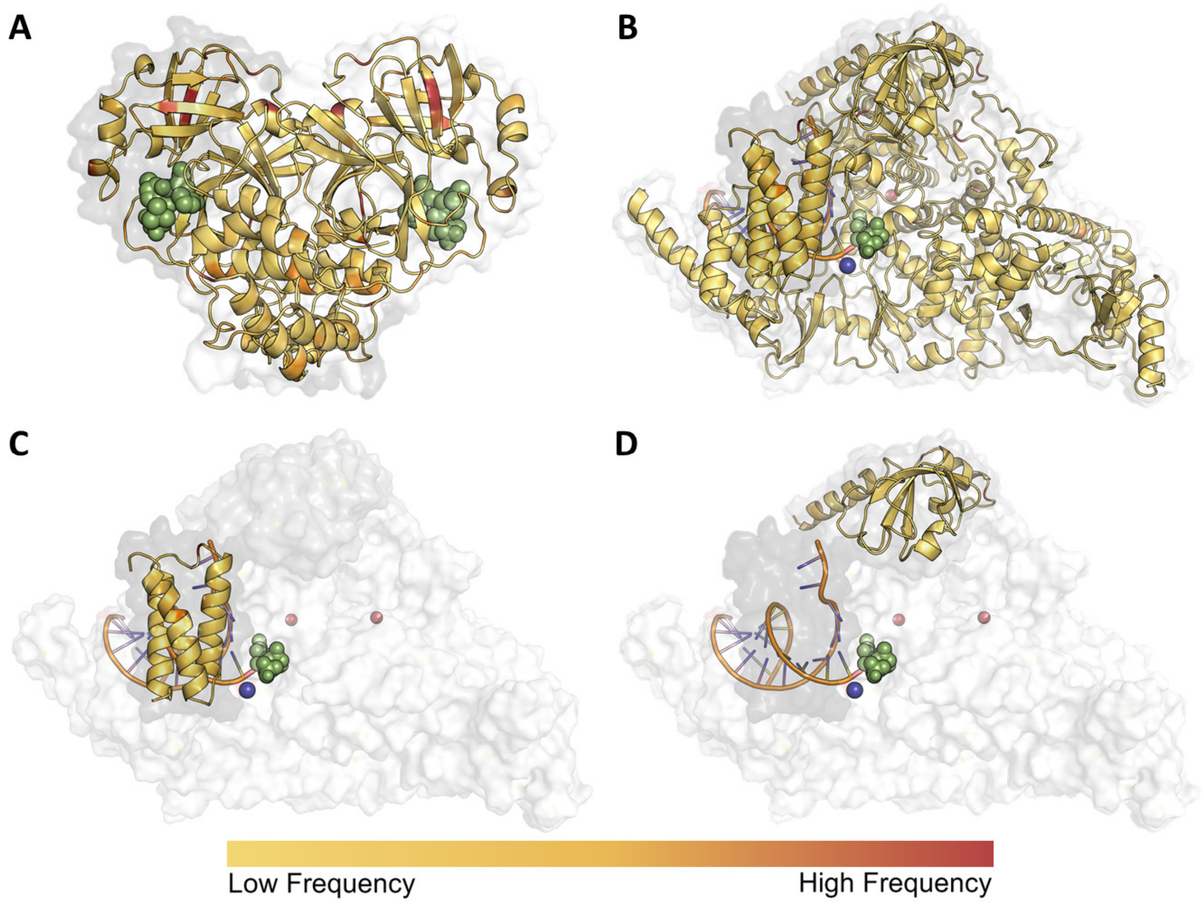

Supplementary Figure S2. Mutation frequency distribution across gene targets. Observing the distribution of low (yellow) to high (red) frequency mutations across the MPro dimer (A) highlighted that medium frequency mutations clustered at the protein-protein interface, which could affect dimer formation. Within the RdRp (B), higher frequency mutations can be observed close to nucleic acid binding within NSP7 (C), possibly affecting related affinity.

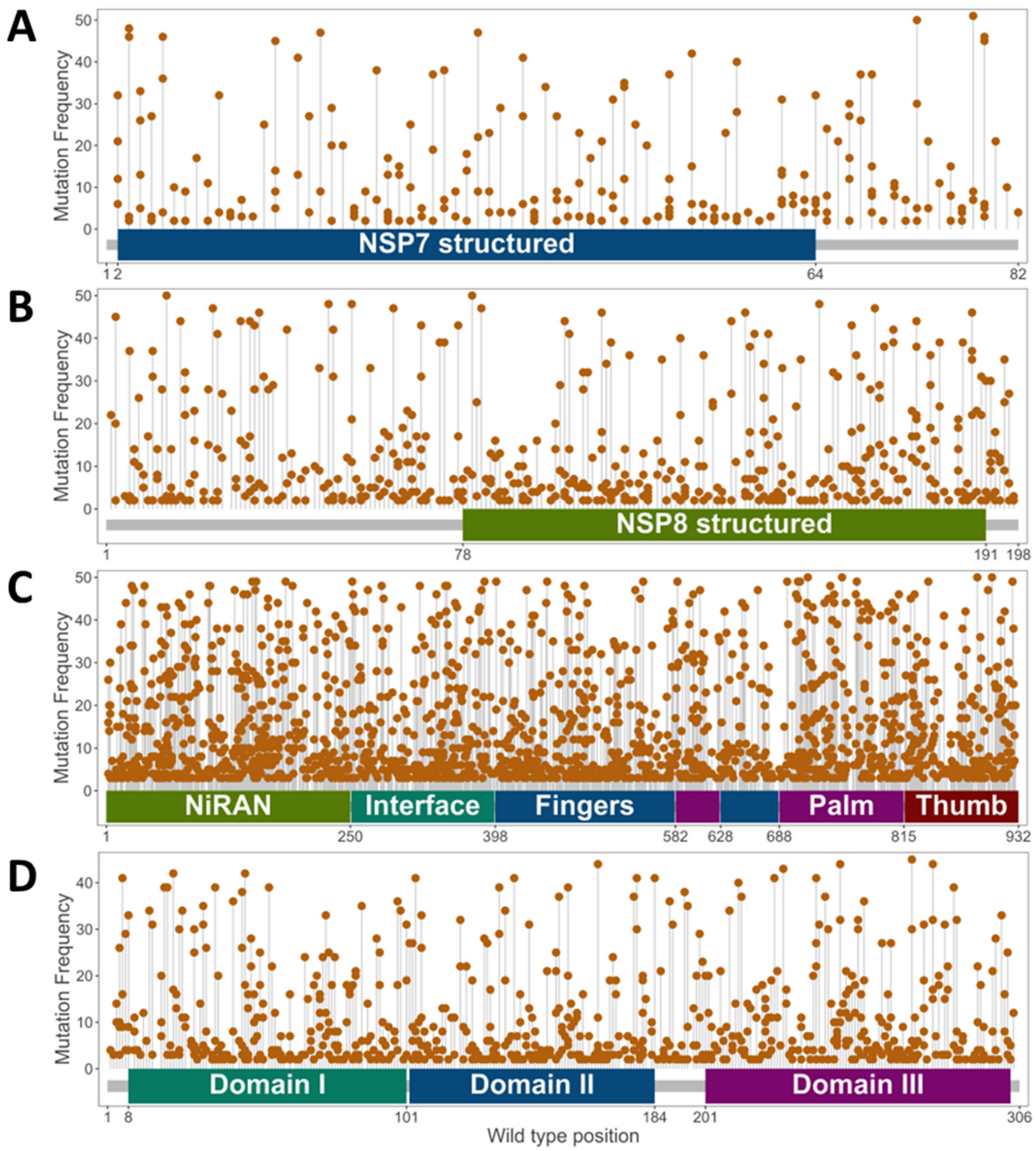

Supplementary Figure S3. Distribution of Medium Frequency mutations across genes. It was observed that medium frequency (Q2-Q3) mutations localized throughout all the genes within the antiviral targets (A-D) and were more abundant within NSP12 (C). Different domains are labelled accordingly, while for NSP7 and NSP8, which constitute one domain each, the crystallized regions within PDB:7BV2 are highlighted.
